# Nectar-dwelling microbes of common tansy are attractive to its mosquito pollinator, *Culex pipiens* L.

**DOI:** 10.1186/s12862-021-01761-5

**Published:** 2021-02-16

**Authors:** D. A. H. Peach, C. Carroll, S. Meraj, S. Gomes, E. Galloway, A. Balcita, H. Coatsworth, N. Young, Y. Uriel, R. Gries, C. Lowenberger, M. Moore, G. Gries

**Affiliations:** 1 Simon Fraser University, 8888 University Drive, Burnaby, BC Canada; 2 The University of British Columbia, 2329 West Mall, Vancouver, BC Canada; 3 University of Saskatchewan, 129-72 Campus Drive, Saskatoon, SK Canada; 4 Emerging Pathogens Institute, University of Florida, 2055 Mowry Road, Gainesville, FL USA

**Keywords:** Mosquitoes, Culex pipiens, Mosquito sugar feeding, Nectar microbes, Microbial volatiles, Insect-microbe interactions

## Abstract

**Background:**

There is widespread interkingdom signalling between insects and microbes. For example, microbes found in floral nectar may modify its nutritional composition and produce odorants that alter the floral odor bouquet which may attract insect pollinators. Mosquitoes consume nectar and can pollinate flowers. We identified microbes isolated from nectar of common tansy, *Tanacetum vulgare*, elucidated the microbial odorants, and tested their ability to attract the common house mosquito, *Culex pipiens*.

**Results:**

We collected 19 microbial isolates from *T. vulgare* nectar, representing at least 12 different taxa which we identified with 16S or 26S rDNA sequencing as well as by biochemical and physiological tests. Three microorganisms (*Lachancea thermotolerans, Micrococcus lactis*, *Micrococcus luteus*) were grown on culture medium and tested in bioassays. Only the yeast *L. thermotolerans* grown on nectar, malt extract agar, or in synthetic nectar broth significantly attracted *Cx. pipiens* females. The odorant profile produced by *L. thermotolerans* varied with the nutritional composition of the culture medium. All three microbes grown separately, but presented concurrently, attracted fewer *Cx. pipiens* females than *L. thermotolerans* by itself.

**Conclusions:**

Floral nectar of *T. vulgare* contains various microbes whose odorants contribute to the odor profile of inflorescences. In addition, *L. thermotolerans* produced odorants that attract *Cx. pipiens* females. As the odor profile of *L. thermotolerans* varied with the composition of the culture medium, we hypothesize that microbe odorants inform nectar-foraging mosquitoes about the availability of certain macro-nutrients which, in turn, affect foraging decisions by mosquitoes.

## Background

Chemical signalling between microbes and insects is widespread and occurs in a variety of contexts [1–3]. Plant odorants as well as visual displays of inflorescences play essential roles in attracting insect pollinators [4]. Floral nectar provides nutrition and habitat for myriad microorganisms [5–10] that may ultimately alter the composition of nectar [11] and produce odorants [8, 12, 13], thereby modifying the inflorescence odor bouquet [13–15]. These microbially-derived odorants may contribute to the plant-pollinator signalling system by serving as attractive semiochemicals (message-bearing chemicals) to pollinators [13, 16–18]. However, this type of signalling may be species-specific with respect to both the sender and the receiver of these semiochemicals because in other instances, microbe-derived odorants cause no behavioral response [19], or avoidance by pollinators [19, 20]. Furthermore, the overall odor bouquet produced by microbes varies with the composition of the microbe community [2, 21], likely modifying the insects’ behavioural responses.

Plant-derived nutrients (e.g., sugars) are fundamental dietary constituents for adult mosquitoes [22], providing energy for flight, mating, blood-feeding, egg-laying, and female overwintering [22–25]. Floral nectar is the dominant source of plant sugar for most mosquitoes but other sugar sources such as extra-floral nectar, aphid honeydew, and fruit juices are also consumed [22, 26]. Inflorescence semiochemicals [27] along with visual inflorescence displays [28] and CO_2_ [29] attract mosquitoes to various inflorescences [22, 29–31] that they distinguish by scent [32, 33] and may pollinate [34–37].

Microbe-derived odorants have not yet been implicated in mosquito nectar-foraging but are exploited by mosquitoes in a variety of other contexts. For example, the odor bouquet of microbe-inoculated or infested aphid honeydew is more attractive to the yellow fever mosquito, *Aedes aegypti,* than the odor bouquet of sterilized honeydew [38]. The volatile semiochemicals emitted by human skin microbes help attract host-seeking mosquitoes [39–41]. Carbon dioxide is another important vertebrate- and plant-host cue for mosquitoes [29, 42], which originates not only from potential hosts but also from their symbiotic microbes [43]. Moreover, microbe-derived semiochemicals indicate suitable oviposition sites for many mosquito species and attract gravid females [44–46]. Microbe-derived semiochemicals and CO_2_ may also play a role in mosquito attraction to floral nectar.

Common tansies, *Tanacetum vulgare*, are visited by many insect taxa [37, 47], including mosquitoes [37, 48] that respond to floral semiochemicals [29] and serve as pollinators [37]. Therefore, *T. vulgare* is a good model plant to investigate the role of nectar-dwelling microbes in the attraction of mosquitoes to floral nectar. Working with *T. vulgare*, and one of its mosquito pollinators, the common house mosquito, *Culex pipiens* [37], we tested the hypotheses (H) that: (1) nectar-colonizing microbes emit semiochemicals attractive to *Cx. pipiens*; (2) the attractiveness of these microbes is dependent upon their nutrient source; and (3) multiple species of nectar-colonizing microbes attract more mosquitoes than a single species.

## Results

### Identification of nectar-colonizing microbes

We collected nectar from nectaries of *T. vulgare* florets with a sterile glass microcapillary tube and identified 19 microbial isolates (Table 1) by sequencing the 16S or 26S rDNA genes, and by comparing the results to data in the National Center for Biotechnology Information GenBank using BLASTn (Bethesda, USA; http://www.ncbu.nlm.nih.gov/BLAST.cgi). We performed additional biochemical and physiological tests on select isolates to aid in their identification (Table 2). Sampling a total of 40 florets from 20 inflorescences (2 florets per inflorescence) from 4 plants (5 inflorescences per plant), we found the yeast *L. thermotolerans* in nectar from two separate florets on two separate plants. *Bacillus* spp. were present in six florets from three separate plants, and *Micrococcus* spp. were present in four florets from three separate plants. In five floret samples, more than one microbe was present.

**Table 1:** (*i*) List of microbes identified from *Tanacetum vulgare* nectar including information about the plant, inflorescence, and individual florets from which they were collected, (*ii*) the medium used to culture microbes, and iii the methods used for microbe identification

			Source				
Isolate	Plate Media	Name of Microbe	Plant #	Inflorescence #	Floret #	ID Method	Accession #
LB-T1D1	LB	Mix of *Bacillus amyloliquefaciens* & *B. subtilis*	1	1	1	PCR + test	MW539056 MW538945
LB-T1E1	LB	*Pseudarthrobacter* sp.	1	2	1	PCR	MW539063
LB-T1E2	LB	*Micrococcus lactis*	1	2	2	PCR + test	MW539682
LB-T2C1	LB	*Pseudomonas* sp.	2	1	1	PCR	MW555319
LB-T2E1	LB	*Staphylococcus epidermidis*	2	1	1	PCR	MW540426
LB-T3A1	LB	*Cryptococcus* sp*.*	3	1	1	PCR	MW555579
LB-T3B2	LB	*Bacillus* sp.	3	2	1	PCR	MW540653
LB-T4A2	LB	*Rhodotorula/Ustilentyloma* sp.	4	1	1	PCR	MW540505
LB-T4B1	LB	*Bacillus circulans*	4	2	1	PCR + test	MW540503
LB-T4B2	LB	*Bacillus* sp.	4	2	2	PCR	MW540792
LB-T4C1	LB	*Micrococcus luteus*	4	3	1	PCR + test	MW540513
LB-T4D1	LB	*Bacillus* sp.	4	3	1	PCR	MW540520
YPD-T1B1	YEPD	*Lachancea thermotolerans*	1	3	1	PCR	MW540525
YPD-T1E1	YEPD	*Micrococcus* sp.	1	2	1	PCR	MW555329
YPD-T2E1	YEPD	*Lachancea thermotolerans*	2	2	2	PCR + test	MW540529
YPD-T3A1	YEPD	*Micrococcus* sp.	3	1	1	PCR	MW555327
YPD-T3B2	YEPD	*Bacillus* sp.	3	2	1	PCR	MW540532
YPD-T4C1	YEPD	*Bacillus* sp.	4	3	1	PCR	MW540612

*LB* Luria–Bertani, *YEPD* yeast extract peptone dextrose, *ID*  identification, *PCR* polymerase chain reaction, *test*  traditional biochemical and physiological tests

**Table 2:** Biochemical tests for the identification of microbes collected from the inflorescences of common tansy, *Tanacetum vulgare*

Test	YPD-T2E1	LB-T1D1-a (round)	LB-T1D1-b (filamentous)	LB-T4B1	LB-T1E2	LB-T4C1
*Fermentation of:*						
Sucrose	+	+	+	+	Not tested	Not tested
Starch	+	+	+	+	Not tested	Not tested
L-alanine	–	–	–	–	Not tested	Not tested
Glucose	+	+	+	+	Not tested	Not tested
Mannitol	–	+	+	+	Not tested	Not tested
Xylose	–	–	–	–	Not tested	Not tested
Galactose	+	–	–	+	Not tested	Not tested
Lactose	–	+	+	Varies	Not tested	Not tested
Fructose	+	+	+	+	Not tested	Not tested
*Enzyme production:*						
Catalase test	+	+	+	+	Not tested	Not tested
Oxidase test	Not tested	–	–	Varies	Not tested	Not tested
Other tests:					Not tested	Not tested
7% NaCl development	Not tested	+	+	–	–	Not tested
10% NaCl development	Not tested	+	+	–	Not tested	Not tested
Nitrate reduction	Not tested	Not tested	Not tested	Not tested	+	+
Hydrolysis of casein	Not tested	Not tested	Not tested	Not tested	+	Not tested
Hydrolysis of tween80	Not tested	Not tested	Not tested	Not tested	Not tested	–
Assimilation of L-arabinose	Not tested	Not tested	Not tested	Not tested	–	Not tested
Assimilation of D-glucose	Not tested	Not tested	Not tested	Not tested	–	Not tested
10 °C development	Not tested	Not tested	Not tested	Not tested	+	–
42 °C development	Not tested	Not tested	Not tested	Not tested	–	Not tested
50 °C development	Not tested	–	+	–	Not tested	Not tested
KOH string test	Not relevant	Gram +	Gram +	Gram +	Not tested	Not tested

**H1**: Nectar-colonizing microbes emit odorants attractive to *Cx pipiens*

**Fig. 1:**
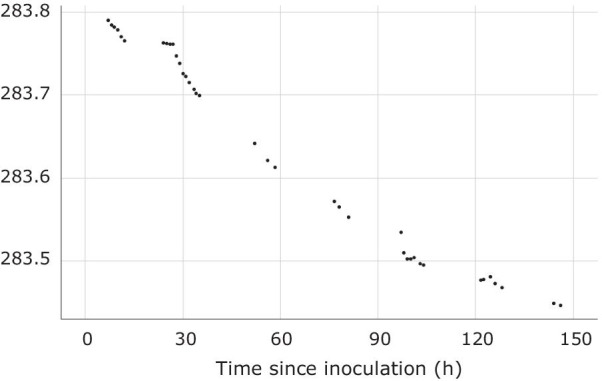
Mean proportion of *Culex pipiens* females captured in paired traps baited with a synthetic nectar broth inoculated, or not (control; light grey bars), with *Micrococcus lactis* (Exp. 1), *Micrococcus luteus* (Exp. 2), or *Lachancea thermotolerans* (Exp. 3). Numbers in white boxes represent the mean percentage of non-responding mosquitoes, and numbers within parentheses the total number of mosquitoes captured. The asterisk (*) in Exp. 3 indicates a significant preference for the treatment stimulus (binary logistic regression with a logit link function, P < 0.05)

In two-choice laboratory experiments with a paired-trap design, we tested attraction of female *Cx. pipiens* to a synthetic nectar broth (10% w/v sucrose, 2% w/v yeast extract) (control stimulus) and the same broth inoculated with (i) *L. thermotolerans* (Exp. 1), (ii) *M. luteus* (Exp. 2), or (iii) *M. lactis* (Exp. 3). We captured more female *Cx. pipiens* when *L. thermotolerans* was the inoculum (z = 4.03, p < 0.0001; Fig. 1, Exp. 1) but not when either *M. luteus* or *M. lactis* was the inoculum (*M. luteus*: z = 1.44, p = 0.15; *M. lactis*: z = − 1.02, p = 0.31; Fig. 1, Exps. 2, 3), indicating an ability of the mosquitoes to discern among different microbes or their metabolites.

### Dose of microbes tested

We grew *L. thermotolerans*, *M. luteus* and *M. lactis* in the synthetic nectar broth for 48 h and these reached final mean concentrations of 3.85 × 10^7^ cells/mL (N = 2), 1.13 × 10^5^ cells/mL (N = 2), and 2.85 × 10^6^ cells/mL (N = 2), respectively.

**H2**: The attractiveness of microbes is dependent upon their growth medium

**Fig. 2:**
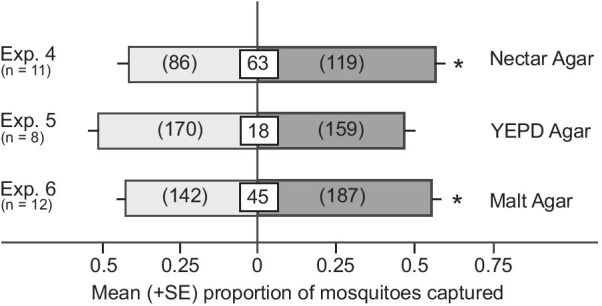
Mean proportion of *Culex pipiens* females captured in paired traps baited with one of three types of media inoculated, or not (control; light grey bars), with *Lachancea thermotolerans.* Numbers in white boxes represent the mean percentage of non-responding mosquitoes, and numbers within the parentheses the total number of mosquitoes captured. The asterisk (*) in Exp. 4 and Exp. 6 indicates a significant preference for the treatment stimulus (binary logistic regression with a logit link function, P < 0.05)

To determine whether the attractiveness of *L. thermotolerans* is affected by its nutrient source, single colonies of *L. thermotolerans* were spread-plated onto synthetic nectar agar, malt extract agar or YEPD agar plates. In two-choice laboratory experiments, we then tested attraction of *Cx. pipiens* to paired traps baited with either one of the three types of inoculated agar (each with 40–60% microbial growth coverage) or an uninoculated control agar. *Lachancea thermotolerans* growing on synthetic nectar agar (Exp. 4) or malt extract agar (Exp. 5) attracted more female *Cx. pipiens* than corresponding agar controls but not when growing on YEPD agar (Exp. 6) (Exp. 4: z = 2.29, p = 0.02; Exp. 5: z = 2.47, p = 0.013; Exp. 6: z = -0.61, p = 0.54; Fig. 2). Thus, attraction of *Cx. pipiens* females to *L. thermotolerans* is contingent upon the nutrients available to this yeast.

**H3**: Multiple species of nectar-colonizing microbes attract more mosquitoes than single microbe species

**Fig. 3:**
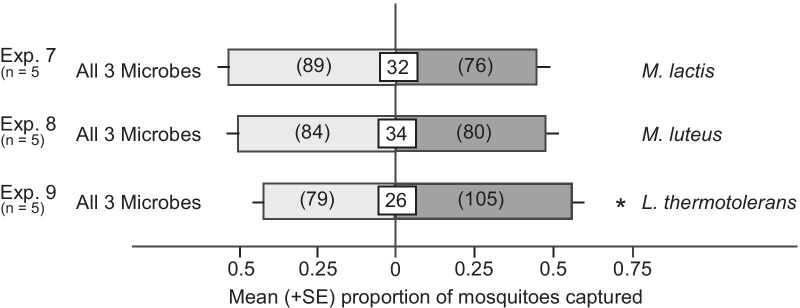
Mean proportion of *Culex pipiens* females captured in paired traps baited with one or three vessels containing synthetic nectar broth, each inoculated with one of three microbes: *Micrococcus lactis, M. luteus* and *Lachancea thermotolerans*. Numbers in white boxes represent the mean percentage of non-responding mosquitoes, and numbers within parentheses the total number of mosquitoes captured. The asterisk (*) in Exp. 9 indicates a significant preference for the *L. thermotolerans* test stimulus (binary logistic regression with a logit link function, P < 0.05)

We hypothesized that odorants from different microbes may have additive or synergistic effects on attraction of *Cx. pipiens* females; therefore, we investigated whether *M. lactis, M. luteus* and *L. thermotolerans* presented together are more attractive than each microbe on its own. We inoculated synthetic nectar in separate petri dishes with single colonies of *M. lactis, M. luteus or L. thermotolerans,* and in laboratory experiments tested attraction of female *Cx. pipiens* to paired traps baited with each species alone or in ternary combination. Surprisingly, the ternary combination was as attractive as *M. lactis* alone (z = − 1.08, p = 0.28; Fig. 3, Exp. 7) and *M. luteus* alone (z = − 0.33, p = 0.74; Fig. 3, Exp. 8), and even less attractive than *L. thermotolerans* alone (z = 1.96, p = 0.05; Fig. 3, Exp. 9). Hence, the attractiveness of *L. thermotolerans* was actually reduced when it was presented alongside the two bacterial species.

### Identification of microbe-derived volatiles

**Fig. 4:**
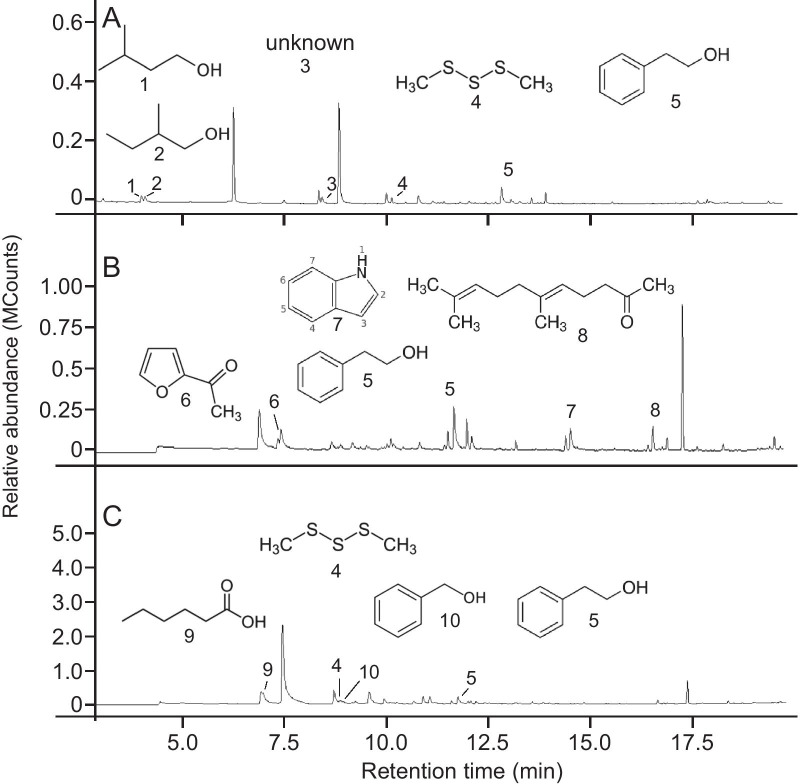
Headspace odorants from plates of YEPD agar (**a**), malt extract agar (**b**), and synthetic nectar agar plates (**c**) that were inoculated with *Lachancea thermotolerans* and that were not present in the headspace of uninoculated control plates. Compounds produced by *L. thermotolerans* were 3-methyl-butanol (1); 2-methyl-butanol (2); unknown (3); dimethyl trisulfide (4); 2-phenylethanol (5); 2-acetyl furan (6); indole (7); geranyl acetone (8); hexanoic acid (9) and benzyl alcohol (10). *Note*: different retention times of the same compounds in panels A-C are due to different temperature programs run during gas chromatographic analyses (see “Methods” for details)

We used dynamic headspace aerations to capture the odorants emitted from *L. thermotolerans* and identified them by gas chromatography-mass spectrometry (GC–MS). In response to the nutrients provided by the three types of media, *L. thermotolerans* produced different odor blends (Fig. 4). The yeast grown on all three media generated 2-phenylethanol, and dimethyl trisulfide was detected in two media types. All microbe-produced compounds differed from those originating from the media themselves (Table 3).

**Table 3:** Headspace odorants of YEPD agar, malt extract agar and synthetic nectar agar, each inoculated with *Lachancea thermotolerans*. Compounds in bold font were found in two or more samples

Medium	Compounds^a^
YEPD agar	2-Methyl-1-butanol
	3-Methyl-1-butanol
	**2-Phenylethanol** **Dimethyl trisulfide**
Malt extract agar	2-Acetylfuran
	**2-Phenylethanol** Indole
	Geranyl acetone
Synthetic nectar agar	Hexanoic acid
	**Dimethyl trisulfide**
	Benzyl alcohol
	**2-Phenylethanol**

^a^All compounds were absent from the headspace of corresponding uninoculated control agar

### CO_2_ production from synthetic nectar

**Fig. 5:**
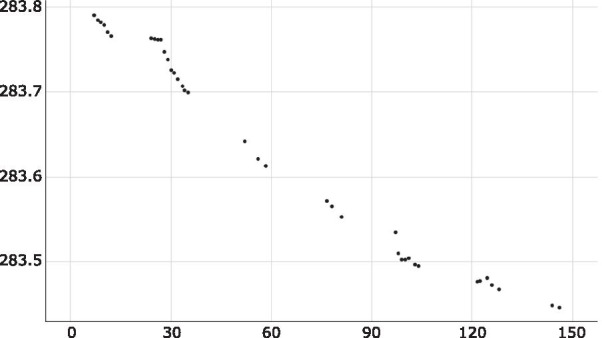
Weight loss over time of synthetic nectar broth (25 mL) inoculated with *Lachancea thermotolerans* due to fermentation and CO_2_ emission by *L. thermotolerans*

Over the course of 150 h, *L. thermotolerans* growing in synthetic nectar broth sealed with a 98% sulfuric acid vapour lock produced 343 mg of CO_2_ (Fig. 5).

## Discussion

Our data show that diverse microbes including the yeast *Lachancea thermotolerans* colonize floral nectar of tansy. *Lachancea thermotolerans* growing on synthetic nectar or malt extract media produce odorants that attract female *Culex pipiens*. Furthermore, we show that *L. thermotolerans* grown in a synthetic nectar broth produces CO_2_ and that *L. thermotolerans* alone is more attractive to female *Cx. pipiens* than when presented along with two bacteria also isolated from the same inflorescences. Below we elaborate on our conclusions.

Our culture-based approach to isolate nectar-colonizing microbes from common tansy likely under-represents the microbial diversity present in nectaries [49]; nevertheless, our collection of 19 isolates representing at least 12 species of microbes compares favourably with results of other studies examining microbial diversity in floral nectar using culture methods [6–8]. We isolated *Staphylococcus epidermidis* which may have been a contaminant because this microbe is typically associated with human skin. However, other microbes, such as *Candida albicans* [50] (not isolated in our study) which are thought to be obligate commensals of animals, have also been isolated from the environment [51]. Although culture-independent methods such as metagenomic analysis can be used to examine microbial diversity without the selection pressures of culturing, only by obtaining live cultures of the colonizing microbes could we study the odor profiles of these isolates and test their ability to attract *Cx. pipiens.*

Microbe-derived semiochemicals have been shown to guide foraging behavior of mosquitoes in a variety of contexts. Semiochemicals emitted from human skin microbiota, including *S. epidermidis*, *Corynebacterium minutissimum* and *Brevibacterium epidermidis*, attract mosquitoes to human hosts [39, 41, 52, 53]. Moreover, semiochemicals from *Psychrobacter immobilis*, *Sphingobacterium multivorum*, *Bacillus* spp., *Pseudomonas* spp., *Klebsiella* spp., and others help mosquitoes locate suitable oviposition sites [44, 46]. Finally, semiochemicals emitted from microbes colonizing aphid honeydew [38], or present in floral nectar [this study], attract sugar-foraging mosquitoes.

Microbes could also inform mosquitoes about the prospect of obtaining a sugar meal. The odor profiles of inflorescences differ not only between plant species [54] but also within the same species due, in part, to microbe-specific odorants [17]. These odorants may enable mosquitoes to discern inflorescences with sugar-rich and sugar-poor rewards, analogous to mosquitoes selecting human hosts based, in part, on their skin microbiota [40, 41]. Emission of microbe-semiochemicals from nectaries could inform mosquitoes about the presence of nectar-dwelling microbes which, in turn, would signal sugar or amino acid metabolism and thus the availability of sugar or amino acids. Alternatively, microbe odorants could indicate microbe presence stemming from a previous insect floral visit that may have temporarily depleted the sugar resource. Mosquitoes themselves are capable of microbe phoresis, as shown with floral nectar surrogates [55], as are many other insects visiting inflorescences or obtaining floral nectar [56, 57].

The informative value of microbe-derived odorants to foraging mosquitoes became evident when we grew *L. thermotolerans* in/on different nutrient sources. The nutrients available to *L. thermotolerans* not only affected the odorants it produced (Fig. 4) but also their attractiveness to foraging mosquitoes. This implies that the presence and composition of specific microbial odorants could inform mosquitoes about the availability of particular nutrients such as carbohydrates and amino acids. *Lachancea thermotolerans* grown in synthetic nectar broth produced appreciable amounts of CO_2_ which—while only weakly attractive on its own [42]—synergistically enhances the attractiveness of floral and host semiochemicals to both nectar- and host-foraging mosquitoes [38, 42].

*Lachancea thermotolerans* and its odor bouquet, respectively, also attract several species of North American yellowjackets (Hymenoptera: Vespidae) [58, 59] and the green lacewing, *Chrysoperla comanche* (Stephens) [60]. When grown and aerated on grape juice agar, *L. thermotolerans* produced 20 odorants which, when field-tested as a synthetic blend, attracted Western yellowjackets, *Vespula pensylvanica* [58]. Two of the odorants in this blend, 2-phentylethanol and 2-acetylfuran, were also found in this study. *Lachancea thermotolerans* is frequently isolated from fruit or fruit-related resources [61, 62] and is used in wine fermentations to generate ethanol and lactic acid [63].

Most studies investigating relationships between nectar-dwelling microbes and floral visitation by insects have focussed on hymenopterans (but see [64]), yet dipterans are also frequent visitors and important pollinators of flowers [65–68], and they interact with microbes [3, 69]. Our results suggest microbe-mediated, or at least modulated, inflorescence visitation by mosquitoes. This concept has been suggested for some hymenopteran pollinators including the European honey bee, *Apis mellifera* [13], and several species of bumble bees, *Bombus* spp. [17, 18], that preferentially visit inflorescences with nectar-dwelling yeasts, primarily *Metschnikowia reukaufii*. Conversely, both honey bees and bumble bees avoid inflorescences with certain nectar-dwelling bacteria [19, 20]. The combined information indicates that microbes can alter the floral scent [15], thereby prompting attraction or avoidance of specific floral visitors.

In follow-up studies, the sample size, while adequate, could be increased and the complete visitation history of plants could be tracked, thereby revealing the insect vectors of any microbe inoculum. Moreover, to determine the amount of inoculum and the resulting odor intensity needed to attract mosquitoes, a dose–response experiment could be run with one test stimulus reflecting the odor intensity of a representative tansy inflorescence. We tested 10 ml of synthetic nectar in our laboratory bioassays. This took into account that (i) each common tansy possesses multiple inflorescences, (ii) each inflorescence comprises dozens of composite flowers, and (iii) each composite flower contains up to 300 florets, each of which can produce up to ~ 1 µL of nectar (D. P. Pers. Obs.). Therefore, the volume of synthetic nectar we bioassayed approximates the volume of nectar produced by a common tansy or a group of common tansies. Similarly, the amount of synthetic nectar odorants we assayed resembled that of odorants produced by nectar-dwelling microbes and which would be encountered by foraging mosquitoes in field settings.

## Conclusion

We demonstrate that floral nectar of common tansies contains various microbes, and we identified 19 to genus level or further. Of the three species tested in behavioral bioassays, only the yeast *L. thermotolerans* had a significant effect on the attraction of female *Cx. pipiens,* which was diminished, rather than improved, by admixture with two bacterial species. The attractiveness of *L. thermotolerans* to *Cx. pipiens* females was dependent upon its nutrient source and linked to a distinct odorant profile, although a causal relationship was not tested. We propose that specific components of the odor blend signal the availability of certain macro-nutrients such as sugar and amino acids which, in turn, inform foraging decisions by mosquitoes. It would now be interesting to determine the key odorant(s) in the microbe odorant blend that mediate(s) the attraction of mosquitoes.

## Methods

### Microbe collection

We collected 40 T*. vulgare* florets, from 20 inflorescences (2 florets per inflorescence), from 4 plants (5 inflorescences per plant) during September, 2017, in Delta, BC (Canada), wearing latex gloves (VWR International, Radnor, USA) and a surgical mask (Acklands Ltd., Wawa, Canada). All plants were collected within a 3-m^2^ patch of tansy beside a secondary road in a rural farming area. Immediately prior to sample collections, DP observed an adult female mosquito (likely *Aedes dorsalis*) visiting this tansy patch. We immediately placed inflorescences into sterile Ziploc bags (S.C. Johnson, Racine, USA) and stored them on ice for transport to the lab. Using ethanol and flame-sterilized scissors, we removed the tops of florets and inserted an autoclaved glass micro-capillary, prepared with a micropipette puller (Model P-1000, Sutter Instrument Co., Novato, USA), into the nectary to draw nectar via capillary action. Each draw yielded a maximum of ~ 1 µL of nectar. We repeated this twice on the same composite flower, using only one composite flower per inflorescence. We then inserted, and subsequently shattered, the micro-capillary into a sterile microcentrifuge tube (1.5 mL; ThermoFisher Scientific Inc., Waltham, USA) containing autoclaved distilled water (400 μL). We pipetted a 100-μL aliquot of this solution onto yeast extract peptone dextrose agar (YEPD) plates (20 g/L bacteriological peptone, 10 g/L yeast extract, 20 g/L glucose, 15 g/L agar) (Sigma Aldrich, St. Louis, USA) and Luria–Bertani agar (LB) plates (10 g/L tryptone, 5 g/L yeast extract, 5 g/L NaCl, 15 g/L agar) (Sigma Aldrich, St. Louis, USA), spread it with a sterile glass rod, and then incubated plates for 48–72 h at 30 °C. We subsequently re-streaked morphologically distinct colonies onto new plates to obtain pure colonies. Working cultures were maintained at 4 °C. Storage cultures of each isolate were prepared with 20% glycerol and stored at − 80 °C.

### Microbe identification

Cells from a single colony were picked and grown in liquid YEPD or LB media for 24 h at 30 °C, and DNA was extracted according to Rose et al. 1990 [70]. DNA concentrations were estimated using a NanoDrop UV/Vis 2000 spectrophotometer (ThermoFisher Scientific Inc., Waltham, USA). To identify bacteria, we used Taq DNA polymerase (Applied Biological Materials, Richmond, Canada) to amplify the V3-V4 loop of the 16S rDNA gene with the Universal Forward Primer (UniF)—5′-CCTACGGGRBGCASCAG-3′ and the Universal Reverse Primer (UniR)—5′-GGACTACNNGGGTATCTAAT-3′ [71], and to amplify the 26S rDNA gene of yeast, we used the NL1 primer—5′-GCATATCAATAAGCGGAGGAAAAG-3′—and NL4 primer—5′-GGTCCGTGTTTCAAGACGG-3′ [72]. The identity of the colony found to be *L. thermotolerans* was confirmed using the specific primers INT2F (5′-TGGTTTTATTGAAGCCAAAGG-3′) and INT2R (5′-GGGGACCCGGAGATTAATAG-3′) [73]. PCR amplicons were pooled and concentrated using the NucleoSpin Gel and PCR Clean-up kit (Machery-Nagel, Duren, Germany). We sequenced amplicons (Genewiz, South Plainfield, USA) and used the Basic Local Alignment Search Tool (BLAST) [74] to compare the sequenced region of individual isolates with known sequences. A species or genus was determined to be a match if there was at least 95% coverage and 99% identity using BLAST with the sequenced isolate. In addition, we ran biochemical and physiological tests on select isolates (see Table 1) and Gram-tested bacterial colonies on select plates using KOH (Table 2). Biochemical tests were carried out according to established procedures [75, 76]. We determined the ability of isolates to grow at 7% NaCl and 10% NaCl in liquid media, and we tested isolate growth at various temperatures. For unknown *Bacillus* and *Epidermidis* isolates, catalase and oxidase enzyme production tests as well as sucrose, starch, L-alanine, glucose, mannitol, galactose, lactose, and fructose fermentation tests were run. For *Micrococcus* isolates, nitrate reduction, hydrolysis, and assimilation tests were run. We identified test isolates by comparing biochemical test results with data from the bioMérieux api® 50 CHB/E test kit (bioMérieux SA, Lyon, France) and reference papers [77–81].

### Rearing of experimental mosquitoes

We reared *Cx. pipiens* at 23–26 °C, 40–60% RH, and a photoperiod of 14L:10D. We kept mixed groups of males and females in mesh cages (30 × 30 × 46 cm high) and provisioned them with a 10-% sucrose solution ad libitum. The primary author (DP) blood-fed females once per week. For oviposition, gravid females were given access to water in circular glass dishes (10 cm diameter × 5 cm high). We transferred eggs to water-filled trays (45 × 25 × 7 cm high) and sustained larvae with NutriFin Basix tropical fish food (Rolf C. Hagen Inc., Baie-D'Urfe, Canada). We transferred pupae via a 7-mL plastic pipette (VWR International, Radnor, USA) to water-containing 354-mL Solo cups covered with a mesh lid. Using an aspirator, we collected emergent adults and placed them in similar cups, along with a cotton ball soaked in a 10-% sucrose solution.

### Behavioural bioassays

We ran all behavioral bioassays in mesh cages (77 × 78 × 104 cm) which were wrapped in black fabric except for the top, thereby allowing ambient fluorescent light illumination. During bioassays, we kept cages at 23–26 °C, 40–60% RH and a photoperiod of 14L:10D. For each 24-h bioassay, we released 50 virgin *Cx. pipiens* females, 1- to 3-day-old, 24-h sugar-deprived into a cage fitted with adhesive-coated (The Tanglefoot Comp., Grand Rapids, USA) paired delta traps (9 cm × 15 cm) on burette stands spaced 30 cm apart. For each bioassay which ran for about 24 h, treatment and control stimuli were randomly assigned to these traps. At the end of each bioassay the number of mosquitoes in each trap was counted.

### Growth conditions for nectar-derived microorganisms

We grew microbes on YEPD agar plates, malt extract agar plates (3% w/v malt extract, 0.2% w/v peptone, 1.5% w/v agar), and synthetic nectar agar or nectar broth (10% w/v sucrose, 2% w/v yeast extract). All agar plates were 92-mm diam Petri dishes (Sarstedt Inc., Nümbrecht, Germany). The synthetic nectar broth was prepared in an autoclaved 2-L Erlenmeyer flask. After streaking single colonies onto plates or inoculating broth, we incubated cultures for approximately 48–72 h at 23–26 °C and 40–60% RH. We used plates with microbial growth covering 40–60% of the surface area for behavioural bioassays.

### Attractiveness of microbes in synthetic nectar

In two-choice laboratory experiments with a paired-trap design, we tested attraction of female *Cx. pipiens* to microbe-derived odorants. We pipetted 10 mL of the treatment stimulus [synthetic nectar broth inoculated with *L. thermotolerans, M. lactis* or *M. luteus* and incubated as described above] into a sterile 92-mm diam Petri dish which we placed into a randomly assigned delta trap. The paired control stimulus consisted of a sterile synthetic nectar broth (10 mL) presented the same way. We bioassayed the response of female *Cx. pipiens* as described above in “behavioural bioassays”.

### Attractiveness of *L. thermotolerans* growing on different media

In two-choice laboratory experiments with a paired-trap design, we tested attraction of female *Cx. pipiens* to *L. thermotolerans* with 40–60% surface area coverage cultured on YEPD agar, malt extract agar, or synthetic nectar agar prepared as described above. In each bioassay, the corresponding uninoculated agar media served as the paired control stimulus.

### Comparative attractiveness of single- vs multiple-species of microbes

In two-choice laboratory experiments with paired traps, we compared attraction of female *Cx. pipiens* to *L. thermotolerans, M. lactis,* and *M. luteus* presented singly or in ternary combination in the same trap. We cultured each microbe separately in synthetic nectar broth as described above, and pipetted 3.3 mL of each broth into a separate sterile 35-mm Petri dish (Sarstedt Inc., Nümbrecht, Germany). As a result, paired traps were baited with either one or three Petri dishes in each bioassay, the design of which is as described in the “Behavioural bioassay” section.

### Dose of microbes tested

We determined the concentration of microbes used in experiments by performing serial dilutions of microbe-inoculated synthetic nectar broth after incubation at 25 °C for 48 h. Cell density was determined using a hemacytometer.

### Measurement of CO_2_ production by *L. thermotolerans*

We inoculated sterile synthetic nectar broth (25 mL) with a single colony-forming unit of *L. thermotolerans* previously grown on YEPD agar, and then incubated the broth at 30 °C for 5 days. We added an aliquot (10 μL) of this broth to 100 mL of sterile synthetic nectar broth in a 250-mL Erlenmeyer flask, attached a vapour lock (5 mL of 98% sulfuric acid) to maintain vapour pressure and prevent water loss, and obtained the starting weight. We incubated the entire assembly in a water bath kept at 30 °C in the fume hood and monitored weight loss as a proxy for CO_2_ emission [82].

### Dynamic headspace odorant collections

We placed 12 plates with *L. thermotolerans* grown for 48–72 h at 23–26 °C on YEPD, malt agar, or SN media into a Pyrex® glass chamber (34 cm high × 12.5 cm wide). A mechanical pump drew charcoal-filtered air at a flow of 1 L min^−1^ for 24–72 h through the chamber and through a glass column (6 mm outer diameter × 150 mm) containing 200 mg of Porapak-Q™ adsorbent. We desorbed odorants captured on Porapak with 0.5 mL each of pentane and ether. We analyzed 2-µl aliquots of Porapak-Q™ extract by gas chromatography-mass spectrometry (GC–MS), operating a Saturn 2000 Ion Trap GC–MS fitted with a DB-5 GC–MS column (30 m × 0.25 mm i.d.; Agilent Technologies Inc., Santa Clara, USA) in full-scan electron impact mode. To chromatograph the odorants of *L. thermotolerans* on malt extract agar and on synthetic nectar agar, we used a flow of helium (35 cm s^−1^) as the carrier gas with the following temperature program: 50 °C (5 min), 10 °C min^−1^ to 280 °C (held for 10 min). The temperature of the injector port was 250 °C and the ion trap was set to 200 °C. To analyze the odorants of *L. thermotolerans* on YEPD agar, and to reveal very volatile compounds that may have eluded detection using the above temperature program, we retained the same helium flow (35 cm s^−1^) but lowered the initial temperature, running the following temperature program: 40 °C (5 min), 10 °C min^−1^ to 280 °C (held for 10 min). Aliquots of headspace odorant extracts were injected in split mode with a 1:1 split ratio, and the temperature of the injector port and the ion trap were set to 250 °C and 200 °C, respectively. We identified odorants in headspace odorant extracts by comparing their retention indices (RI; relative to *n-*alkane standards) [83] and their mass spectra with those reported in the literature and with those of authentic standards.

### Statistical analyses

We used SAS software version 9.4 (SAS Institute Inc., Cary, USA) to analyze data, excluding from analyses all experimental replicates with no mosquitoes captured in traps. We used a binary logistic regression model with a logit link function to compare mean proportions of responders between test stimuli, and used back-transformed data to attain means and confidence intervals.

## Data Availability

The datasets generated and or analysed during the current study are available in the Dryad repository [https://doi.org/10.5061/dryad.j6q573ncw], and the genetic sequences are available from the National Center for Biotechnology Information GenBank [https://www.ncbi.nlm.nih.gov/genbank] (see Table 1 for accession numbers).

## Abbreviations

GC–MS: Gas chromatography–mass spectrometry
LB: Luria–Bertani medium
PCR: Polymerase chain reaction
RH: Relative humidity
RI: Retention index
YEPD: Yeast extract peptone dextrose medium
