# Correction to: Nectar-dwelling microbes of common tansy are attractive to its mosquito pollinator, *Culex pipiens* L.

**DOI:** 10.1186/s12862-021-01769-x

**Published:** 2021-03-08

**Authors:** D. A. H. Peach, C. Carroll, S. Meraj, S. Gomes, E. Galloway, A. Balcita, H. Coatsworth, N. Young, Y. Uriel, R. Gries, C. Lowenberger, M. Moore, G. Gries

**Affiliations:** 1 Simon Fraser University, 8888 University Drive, Burnaby, BC Canada; 2 The University of British Columbia, 2329 West Mall, Vancouver, BC Canada; 3 University of Saskatchewan, 129-72 Campus Drive, Saskatoon, SK Canada; 4 Emerging Pathogens Institute, University of Florida, 2055 Mowry Road, Gainesville, FL USA

## Correction to: BMC Ecol Evol (2021) 21:29 10.1186/s12862-021-01761-5

Following publication of the original article [1], we were notified that Fig. 1 was mistakenly replaced in Production with a duplicate of Fig. 5. 

The original article has been corrected.
